# Focused ultrasound-mediated blood–brain barrier opening is safe and feasible with moderately hypofractionated radiotherapy for brainstem diffuse midline glioma

**DOI:** 10.1186/s12967-024-05096-9

**Published:** 2024-03-30

**Authors:** Masih Tazhibi, Nicholas McQuillan, Hong-Jian Wei, Matthew Gallitto, Ethan Bendau, Andrea Webster Carrion, Xander Berg, Danae Kokossis, Xu Zhang, Zhiguo Zhang, Chia-Ing Jan, Akiva Mintz, Robyn D. Gartrell, Hasan R. Syed, Adriana Fonseca, Jovana Pavisic, Luca Szalontay, Elisa E. Konofagou, Stergios Zacharoulis, Cheng-Chia Wu

**Affiliations:** 1 Department of Radiation Oncology, Columbia University Irving Medical Center, 622 W. 168th Street, New York, NY 10032 USA; 2 Department of Biomedical Engineering, Columbia University, New York, NY 10027 USA; 3 Division of Pediatric Hematology Oncology and Stem Cell Transplant, Department of Pediatrics, Columbia University Irving Medical Center, 161 Fort Washington Avenue, New York, NY 10032 USA; 4 Department of Pediatrics, Children’s Hospital of Philadelphia, Philadelphia, PA 19104 USA; 5 Institute for Cancer Genetics, Columbia University Irving Medical Center, New York, NY 10032 USA; 6 Department of Pathology and Cell Biology, Columbia University Irving Medical Center, New York, NY 10032 USA; 7 Department of Pathology and Laboratory Medicine, Kaohsiung Veterans General Hospital, Kaohsiung, 813 Taiwan; 8 Department of Radiology, Columbia University, New York, NY 10027 USA; 9 Division of Pediatric Oncology, Department of Oncology, Johns Hopkins School of Medicine, Baltimore, MD 21287 USA; 10 Department of Neurosurgery, Children’s National Hospital, Washington, DC USA; 11 George Washington University, Washington, DC USA; 12 Center for Cancer and Blood Disorders, Children’s National Hospital, Washington, DC USA; 13 The Brain Tumor Institute, Children’s National Hospital, Washington, DC USA; 14 Bristol Myers Squibb, Princeton, NJ 08901 USA; 15 Herbert Irving Comprehensive Cancer Center, New York, NY 10032 USA

**Keywords:** Focused ultrasound, Radiotherapy, Diffuse midline glioma, Blood–brain barrier opening

## Abstract

**Background:**

Diffuse midline glioma (DMG) is a pediatric tumor with dismal prognosis. Systemic strategies have been unsuccessful and radiotherapy (RT) remains the standard-of-care. A central impediment to treatment is the blood–brain barrier (BBB), which precludes drug delivery to the central nervous system (CNS). Focused ultrasound (FUS) with microbubbles can transiently and non-invasively disrupt the BBB to enhance drug delivery. This study aimed to determine the feasibility of brainstem FUS in combination with clinical doses of RT. We hypothesized that FUS-mediated BBB-opening (BBBO) is safe and feasible with 39 Gy RT.

**Methods:**

To establish a safety timeline, we administered FUS to the brainstem of non-tumor bearing mice concurrent with or adjuvant to RT; our findings were validated in a syngeneic brainstem murine model of DMG receiving repeated sonication concurrent with RT. The brainstems of male B6 (Cg)-Tyrc-2J/J albino mice were intracranially injected with mouse DMG cells (PDGFB^+^, H3.3K27M, p53^−/−^). A clinical RT dose of 39 Gy in 13 fractions (39 Gy/13fx) was delivered using the Small Animal Radiation Research Platform (SARRP) or XRAD-320 irradiator. FUS was administered via a 0.5 MHz transducer, with BBBO and tumor volume monitored by magnetic resonance imaging (MRI).

**Results:**

FUS-mediated BBBO did not affect cardiorespiratory rate, motor function, or tissue integrity in non-tumor bearing mice receiving RT. Tumor-bearing mice tolerated repeated brainstem BBBO concurrent with RT. 39 Gy/13fx offered local control, though disease progression occurred 3–4 weeks post-RT.

**Conclusion:**

Repeated FUS-mediated BBBO is safe and feasible concurrent with RT. In our syngeneic DMG murine model, progression occurs, serving as an ideal model for future combination testing with RT and FUS-mediated drug delivery.

**Supplementary Information:**

The online version contains supplementary material available at 10.1186/s12967-024-05096-9.

## Background

Diffuse midline glioma (DMG) is a central nervous system (CNS) tumor with dismal prognosis [1]. While these cancers can emerge in adults, they predominate in children and represent one of the highest rates of brain tumor-related mortality in this population. Median survival is ~ 1 year, with over 90% of patients succumbing to the disease within 2 years [1, 2].

In past decades, various chemotherapeutic agents using different combinations and timing strategies have been explored, yet failed to improve outcomes [3]. Surgical resection plays virtually no role in treating brainstem DMG, given the eloquent location and diffuse topography of these tumors [3–5]. As such, radiotherapy (RT) remains the sole treatment available for this disease. Typically, RT consists of 54 Gy in 1.8 Gy per fraction; however, an acceptable alternative is 39 Gy in 13 fractions (39 Gy/13fx) [6]. Regardless, RT confers a survival benefit of ~ 3 months, although disease progression is inevitable [6].

The enduring difficulty limiting success of systemic therapies in brainstem DMG is the blood–brain barrier (BBB), which protects the brain from neurotoxicity yet precludes drug delivery to the site of malignancy [7, 8]. In recent years, several treatment modalities have attempted to circumvent this limitation with nominal success, including high-dose chemotherapy, intra-arterial agents, and convection-enhanced delivery [9–13]. Focused ultrasound (FUS) with microbubbles (MBs) provides one compelling solution, transiently and non-invasively disrupting the BBB to enhance drug delivery [7, 14]. Preclinical efforts have been especially rewarding with this approach, as FUS has been shown to increase the parenchymal penetrance of various agents in the brain and increase survival in mice harboring primary cerebral tumors without producing long-term tissue damage [14–22]. Recent work has also established the feasibility of FUS-enhanced drug delivery at the brainstem in rodents with and without brainstem tumors [16, 23–25]. These efforts have inspired several clinical studies in humans, including a trial in children with progressive DMG [NCT04804709].

While the future looks promising for FUS-based treatment paradigms, the necessary question for clinical translation of this technology for DMG is whether its use is safe and feasible in combination with RT, the true standard-of-care for this disease. We hypothesized that FUS-mediated blood–brain barrier opening (BBBO) with RT would be safe and feasible. To test this hypothesis, we administered FUS to the brainstem either adjuvant to or concurrent with a clinical RT regimen.

## Materials and methods

### Animal studies

Animal protocol AC-AABP4566 was compliant with ARRIVE guidelines and approved for this research by the Columbia University Institutional Animal Care and Use Committee (IACUC). B6 (Cg)-Tyrc-2J/J mice (B6-albino), 6–9 weeks old, were acquired from Jackson Laboratories (Bar Harbor, ME) for this study (n = 51).

### Experimental design

**Fig. 1:**
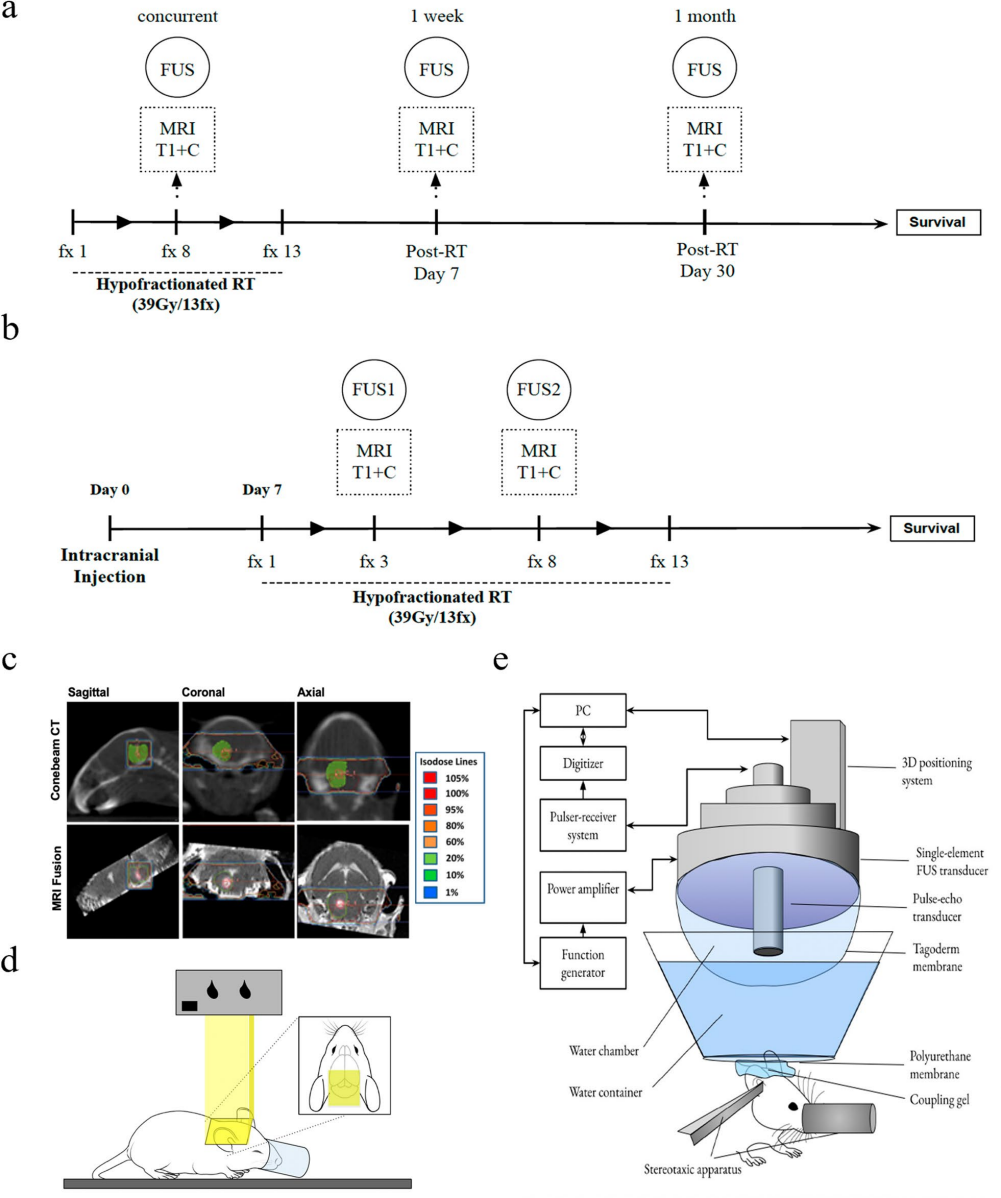
Experimental design, radiotherapy, and FUS delivery. **a** Schematic diagram of experimental timeline of non-tumor bearing mice. RT was delivered with 39 Gy/13fx Monday-Friday over 2.6 weeks. In RT + FUS combination groups, animals received one round of FUS either 1 month after, 1 week after, or concurrently with RT. **b** Schematic diagram of experimental timeline of tumor-bearing mice. 39 Gy/13fx of RT was delivered starting from 1 week after tumor implantation. RT + FUS animals underwent two rounds of FUS spaced 1 week apart concurrently with RT. T1-weighted contrast-enhanced (T1 + C) MRI was obtained after each sonication to confirm BBBO. **c** Representative images of MRI-based radiation treatment planning of SARRP. The green contour indicates the T2 hyperintensity of the tumor with approximately 1 mm expansion respecting anatomical boundaries. The isodose lines were shown in different colors representing the percentage of prescription dose delivered. **d** Schematic diagram of the experimental setup RT with XRAD-320. The yellow column represents the RT delivered through 2 × 2 cm^2^ collimator designed in an axial arrangement. **e** Schematic diagram of the experimental setup for FUS-induced BBB opening

Our study was split into two phases. In phase one, we assessed safety and feasibility of FUS-mediated BBBO in non-tumor-bearing mice at three time points: 1 month after RT, 1 week after RT, and concurrent with RT (Fig. 1a). Phase two involved validating safety and feasibility in tumor-bearing mice undergoing concurrent RT (Fig. 1b). To replicate a clinical RT regimen, 39 Gy/13fx was delivered to the brainstem Monday-Friday over 2.6 weeks. To assess potential impacts of FUS on motor function, animals underwent Kondziela’s inverted screen testing and Deacon sequential weight lifting [26] before and after sonication. Furthermore, cardiopulmonary function was assessed throughout each sonication. In non-tumor-bearing mice, physiological and body weight monitoring was performed for one month following treatment cessation. In tumor-bearing mice, animals underwent daily inspection and weekly non-contrast T2-weighted magnetic resonance imaging (MRI) to monitor tumor growth. Kaplan–Meier survival analysis was performed once animals met either condition for termination: > 20 percent weight loss, significant neurological deficit, or death. In separate experiments, both non‐tumor‐and tumor‐bearing animals underwent RT, with or without FUS, followed by histological analysis to assess for pathological signs of inflammation and tissue damage.

### Cell line

To generate a murine syngeneic xenograft model of brainstem DMG, we used 4423 DMG cells [27]. The cells were cultured in suspension using NeuroCult™ Basal Medium (STEMCELL Technologies, Vancouver, BC, Canada) with 10% NeuroCult™ Proliferation Supplement (STEMCELL Technologies, Vancouver, BC, Canada), 100 units/mL penicillin, 100 μg/mL streptomycin, 20 ng/mL human basic FGF, 10 ng/mL human EGF, and 2 μg/mL heparin, and were incubated at 37 °C with 5% CO_2_.

### Intracranial implantation

To establish the syngeneic brainstem murine model of DMG, mice were anesthetized with 1–2% isoflurane and immobilized in a stereotaxic instrument (Stoelting, Wood Dale, IL, USA). A 1-cm incision was made at the scalp midline to expose the sagittal suture and lambda. A burr hole 1 mm in diameter was made approximately 1.5-mm posterior to the lambda and 1.5-mm lateral to the sagittal suture. A Hamilton syringe containing 100,000 cells suspended in 1 μL NeuroCult™ Basal Medium (STEMCELL Technologies, Vancouver, BC, Canada) was inserted 5.5-mm below the skull surface and injected at a rate of 0.1 μL/min over 10 min. Two minutes following implantation, the syringe was removed from the mouse brain in 1 mm increments spaced 30 s apart [17].

### Magnetic resonance imaging and image analysis

A 9.4 T MRI system (Bruker Medical, Boston, MA, USA) was utilized for verification of tumor growth and BBBO. Mice were anesthetized and placed into a birdcage coil (diameter 35-mm). To validate tumor growth, T2-weighted images were obtained using a T2-weighted TurboRARE sequence. To confirm BBB opening/closure, contrast-enhanced T1-weighted images were acquired using a T1-weighted 2D FLASH sequence following intraperitoneal injection of 0.2 mL gadodiamide (GD-DTPA) (Omniscan, GE Healthcare, Princeton, NJ, USA). The free, open-source platform 3D Slicer (www.slicer.org) was used to quantify BBBO and tumor volume.

### Radiotherapy

The Small Animal Radiation Research Platform (SARRP) was the primary modality for RT with the XRAD-320 as backup. We used the SARRP (Xstrahl, Suwanee, GA, USA) to conduct MRI-guided RT. First, cone-beam computed tomography (CBCT) was obtained from mice anesthetized under 1–2% isoflurane anesthesia using the onboard scanner of the SARRP. CBCT images were registered and fused with T2-weighted MRI DICOM images by using the MuriPlan preclinical treatment planning system (Xstrahl, Suwanee, GA, USA). The tumor was identified and contoured from the T2-weighted image. We contoured the tumor with approximately 1 mm expansion of its T2 hyperintensity respecting anatomical boundaries (Fig. 1c, green contour). Two beams were designed in opposite sagittal arrangements to deliver 3 Gy radiation through a 5 × 5 mm^2^ collimator prescribed to the isocenter at the brainstem (non-tumor-bearing mice) or tumor contour (tumor-bearing mice) (Fig. 1c). For the XRAD-320 Biological Irradiator (Precision X-Ray Inc, Madison, CT, USA), mice were anesthetized under 1–2% isoflurane and positioned within the targeting range of an adjustable collimator designed in an axial arrangement. To deliver 3 Gy radiation at a depth of 5 mm (i.e., at the brainstem), the following settings were used: 320 kV, 12.5 mA, 86 s exposure time, SSD of 50 cm, 2 mm Al filter, and 2 × 2 cm^2^ collimator size (Fig. 1d).

### Focused ultrasound (FUS)

The experimental and technical setup of FUS is outlined in Fig. 1e and has been previously described [17]. A single-element, spherical-segment FUS transducer was driven by a function generator through a 50-dB power amplifier. A single-element, pulse-echo transducer was housed within the central core of the FUS transducer and used for passive cavitation detection (PCD) of acoustic emissions. PCD signals were segmented into stable harmonic cavitation dose (SCD_h_), stable ultraharmonic cavitation dose (SCD_u_), and inertial cavitation dose (ICD) by analyzing the signal in the frequency-domain and filtering the harmonic, ultraharmonic, and broadband spectral areas [28]. In-house manufactured MBs were injected intravenously and FUS transducer was applied at the brainstem ~ 1.5 mm lateral to the midline to spare the basilar artery. Sonication was delivered at 0.5 MHz with a peak-negative pressure of 0.3 MPa in bursts of 10 ms length at 5 Hz repetition time over 120 s (600 pulses).

To evaluate potential cardiopulmonary abnormalities due to FUS, all treatment animals underwent continuous monitoring of vitals before, during, and after sonication. A Biopac pressure pad with a respiratory transducer (Biopac Systems, Inc., CA, USA) was placed below the mouse and Biopac ECG leads were attached to the extremities. Within an hour following sonication, BBBO, as well as signs of acute hemorrhage, were assessed using T1-weighted contrast-enhanced (T1 + C) MRI.

### Motor and coordination testing

To assess brainstem-related strength and coordination, all animals underwent Kondziela’s inverted screen testing and Deacon sequential weightlifting [16, 26] 60 min before FUS and 60 min after recovery from anesthesia.

### Histology

To evaluate potential tissue damage associated with FUS use combined with RT, a subset of mice in each group underwent cardiac perfusion within three days of their last treatment session. Brains were collected and fixed in a 10% formalin solution. Hematoxylin and eosin (H&E) staining was performed, and the slides were analyzed by a blinded neuropathologist (C.I.J.). Intraparenchymal injury, including degree of brainstem hemorrhage and inflammation, was qualitatively compared between groups.

### Survival analysis

Animals selected for survival were inspected daily for changes indicative of brainstem injury and/or disease progression. Primary endpoints included: animal death, weight loss exceeding 20% of initial body weight, and any indication of serious illness (i.e., hunched posture, labored breathing).

### Ethical approval

This study adhered to all institutional guidelines for proper care and use of animals, as outlined by the Institutional Animal Care and Use Committee (IACUC) and was completed in accordance with the ARRIVE guidelines.

## Results

### PCD and BBBO confirmation

**Fig. 2:**
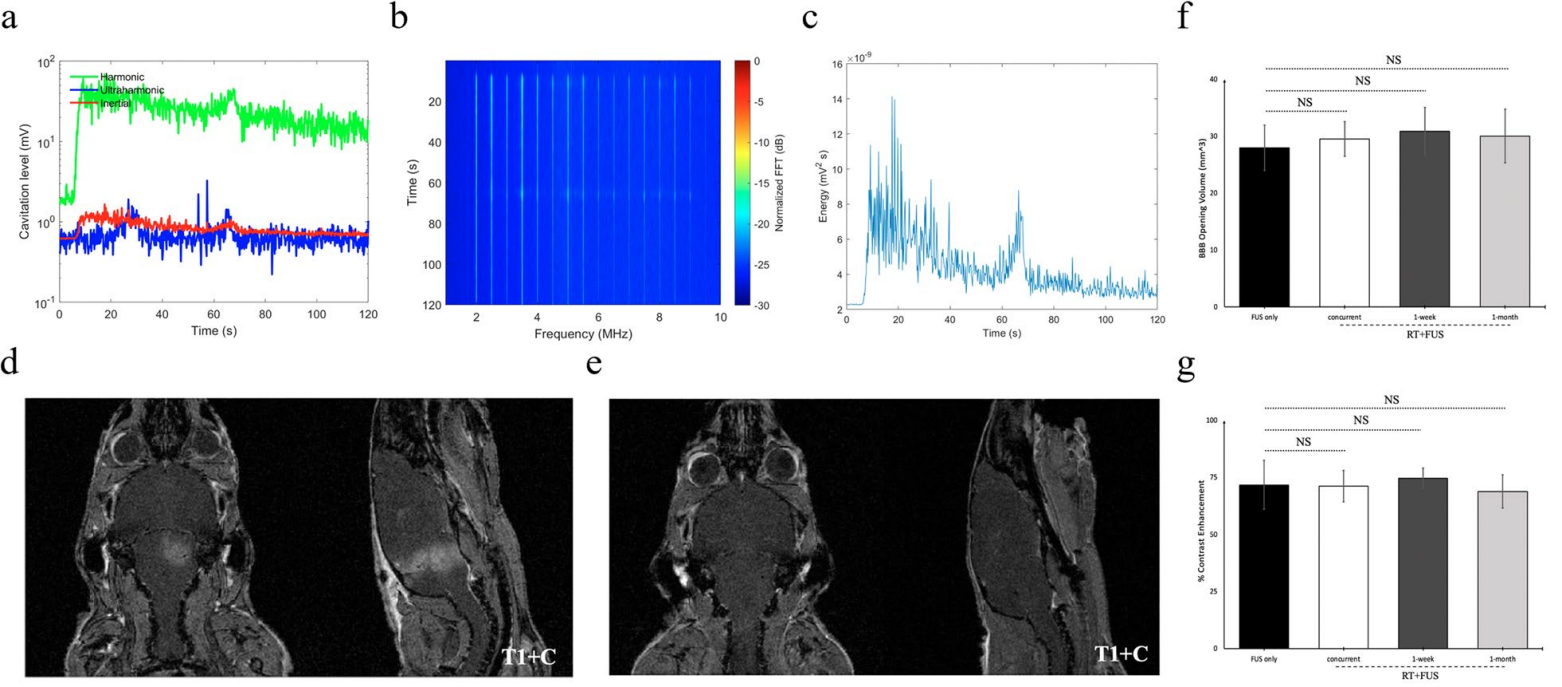
BBB disruption, passive cavitation detection, and in vivo quantification. Representative in vivo passive cavitation detection measurements. **a** The doses of stable harmonic cavitation (green), stable ultraharmonic (blue), and inertial cavitation (red) throughout sonication. **b** Spectrogram and **c** acoustic energy of MBs cavitation during FUS exposure. **d** Representative T1 + C MRI confirmed BBBO after FUS sonication, with **e)** BBB closure observed approximately 72 h later. The quantification of T1 + C MRI for (**f**) BBB opening volume (mm^3^) and (**g**) contrast enhancement (%). Values are group means ± SD. P-values were calculated relative to the FUS-only group using unpaired t tests with Welch’s correction. NS indicates non-significant (P > 0.05)

BBBO was confirmed for all non-tumor-bearing mice receiving brainstem FUS. During sonication, PCD was used in real time to detect the acoustic emission of MBs. Representative PCD results are shown in Fig. 2a–c. MB injection caused a 30-fold increase in SCD_h_ (green curve, Fig. 2a), but not SCD_u_ (blue curve, Fig. 2a) or ICD (red curve, Fig. 2a), relative to baseline. SCD_h_ increased to approximately one order of magnitude greater than both SCD_u_ and ICD and dominated the emissions spectrogram of the treatment window (Fig. 2b). Acoustic energy emitted by MBs was greater at treatment initiation and gradually declined as microbubbles were cleared from the cerebrovascular system (Fig. 2c). Taken together, SCDh, SCDu, and ICD were relatively constant throughout the sonication, suggesting persistent stable cavitation activity during sonication with minimal inertial cavitation. T1 + C MRI confirmed BBBO for all treatment mice, with closure observed 72 h post-sonication. Representative images are shown in Fig. 2d, e. Quantitative analysis of T1 + C MRI for BBBO volume (mm^3^) showed no significant difference between FUS-only (mean = 27.98 ± 3.81) and RT + FUS treatment groups (concurrent, mean = 29.51 ± 2.90; 1-week, mean = 30.87 ± 4.02; 1-month, mean = 30.05 ± 4.51) (Fig. 2f). Likewise, comparison of contrast enhancement (%) showed no significant difference between FUS-only (mean = 71.77 ± 10.70) and RT + FUS groups (concurrent, mean = 71.26 ± 6.57; 1-week, mean = 74.66 ± 4.18; 1-month, mean = 68.90 ± 6.98) (Fig. 2g).

### Safety and feasibility of BBBO and RT in non-tumor-bearing mice

**Fig. 3:**
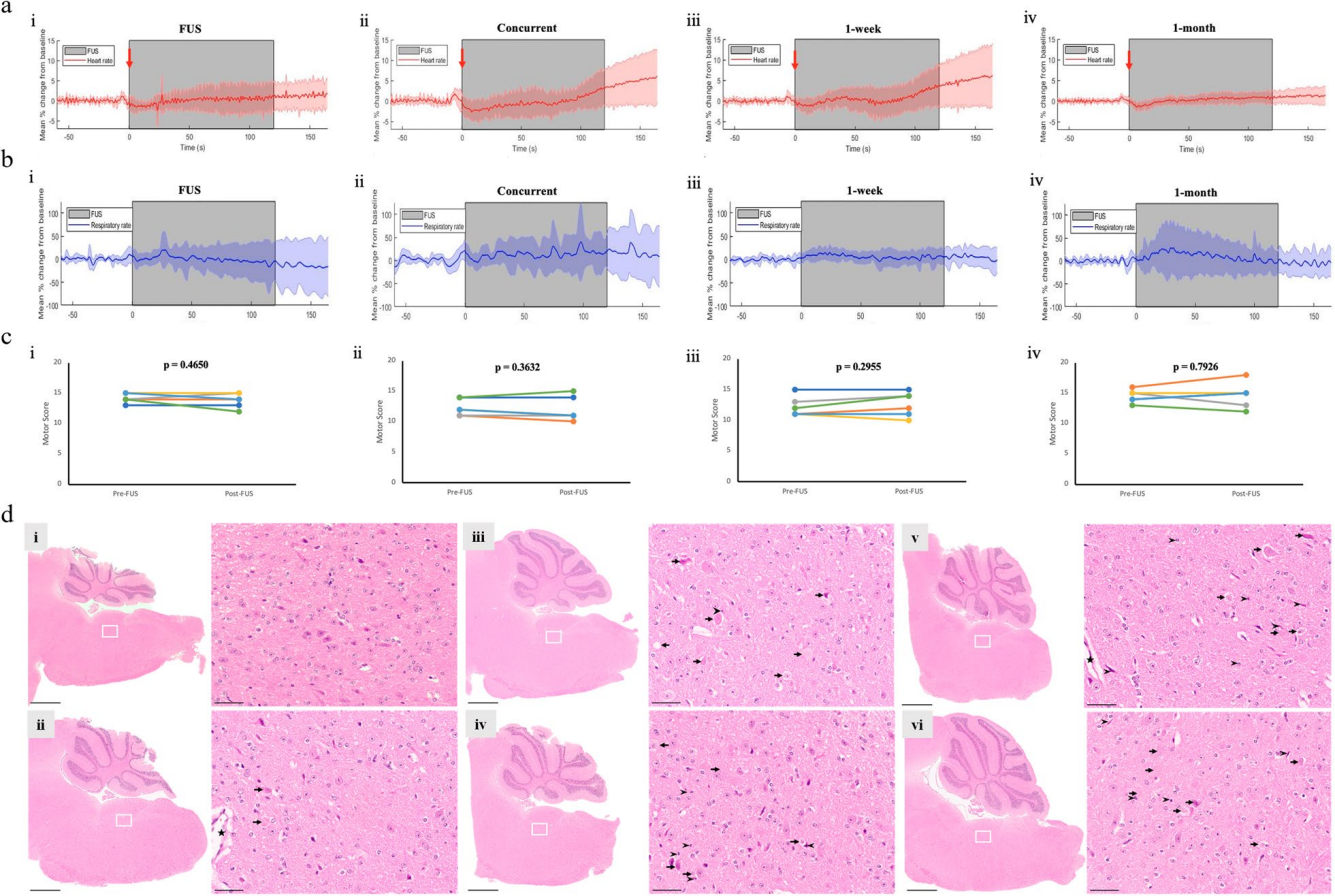
Cardiopulmonary vitals, motor testing, and tissue integrity. Non-tumor bearing mice (n = 6) (**a**) heart and (**b**) respiratory rates were measured before, during, and after brainstem sonication. Red arrows indicate timepoint of MBs injection, grey regions depict timepoint of FUS treatment window, solid horizontal lines indicate group means, and surrounding shaded areas represent one standard deviation. **c** Deacon sequential weightlifting test. i: FUS-only, ii: RT + FUS concurrent, iii: RT + FUS 1-week, iv: RT + FUS 1-month. Paired motor data for each mouse is represented in different colors for ease of visualization. **d** Representative H&E staining of brainstem tissues from control (i), FUS-only (ii), RT-only (iii), RT + FUS concurrent (iv), RT + FUS post 1-week (v), and RT + FUS post 1-month (vi) groups. Each right panel is magnified from the white square of the left panel. Long arrows indicate cell swelling, vacuolar degeneration, and eosinophilic neurons with pyknosis. Arrow heads indicate mononuclear cell infiltration. Filled star: Blood vessel. Scale bar = 1 mm (left panels), 50 µm (right panels)

All non-tumor-bearing mice in the FUS and RT + FUS groups underwent cardiorespiratory monitoring throughout sonication. Recording began 45 s prior to FUS to establish a baseline and continued until 45 s after completion. Mean changes in cardiac and respiratory rates with standard deviation for FUS-only, concurrent RT + FUS, RT + FUS 1-week, and RT + FUS 1-month are represented in Fig. 3a, b. All groups (i-iv) displayed an injection-associated decline in heart rate which spontaneously recovered (Fig. 3a). This is likely a physiological response to the intravascular fluid bolus. No pathophysiological responses, including cardiac pause, apnea, or significant variation in respiratory rate, were observed. FUS did not cause a significant change in vitals compared to baseline. In addition to the vital functions, the brainstem also plays an important role in regulating motor functions, including locomotion, posture, and balance. Hence, we performed Kondziela’s inverted screen testing and Deacon sequential weightlifting to evaluate the effects of RT and FUS on brainstem-related strength and coordination. In all non-tumor-bearing mice receiving sonication, no behavioral symptoms emerged indicative of brainstem pathophysiology. All mice completed the 60-s Kondziela’s inverted screen test before and after sonication without any changes (data not shown). Deacon sequential weightlifting revealed no significant difference in motor score before and after treatment for all groups (Fig. 3c).

To assess intraparenchymal injury from RT and/or FUS, a blinded neuropathologist (C.I.J.) reviewed H&E-stained tissues from control and treated mice. Compared to the control group (Fig. 3d, i), the FUS-only group showed near-normal morphology of brainstem tissue with no noticeable degenerative neurons (Fig. 3d, ii, long arrows). RT has been shown to potentially induce neuroinflammation and neurodegeneration in the brain. Hence, in all RT groups, we observed only minimal neuronal inflammation, including degenerative (so-called eosinophilic) neurons showing bright eosinophilic cytoplasm, cytoplasmic shrinkage, pyknotic nuclei, and occasional swelling and vacuolation (Fig. 3d iii–vi, long arrows). With the addition of FUS, we found a mild increase in mononuclear cell infiltration (Fig. 3d, iv–vi). Although we cannot confirm the cell types of infiltration without specific marker staining, our group recently reported that FUS-mediated BBB opening increases microglia and CNS-associated macrophage in the brain [29]. Lastly, no intraparenchymal microhemorrhage and tissue necrosis were observed among all groups, indicating either RT, FUS, or combination treatment did not cause any serious parenchymal damage. A subset of non-tumor bearing mice in each group (n = 4) was monitored for one month following treatment cessation for signs of physiological abnormality and weight loss. FUS was well tolerated, and all mice survived the post-treatment monitoring window without illness.

### Safety and feasibility of BBBO and RT in syngeneic DMG murine model

After demonstrating feasibility of brainstem BBBO adjuvant to and concurrent with RT in non-tumor-bearing mice, we assessed safety and feasibility of repeated FUS-mediated BBBO with concurrent hypofractionated RT in mice with brainstem DMG. Tumor-bearing mice underwent either no treatment (n = 6), RT only (n = 9), or two sonication sessions concurrent with RT (n = 7).

**Fig. 4:**
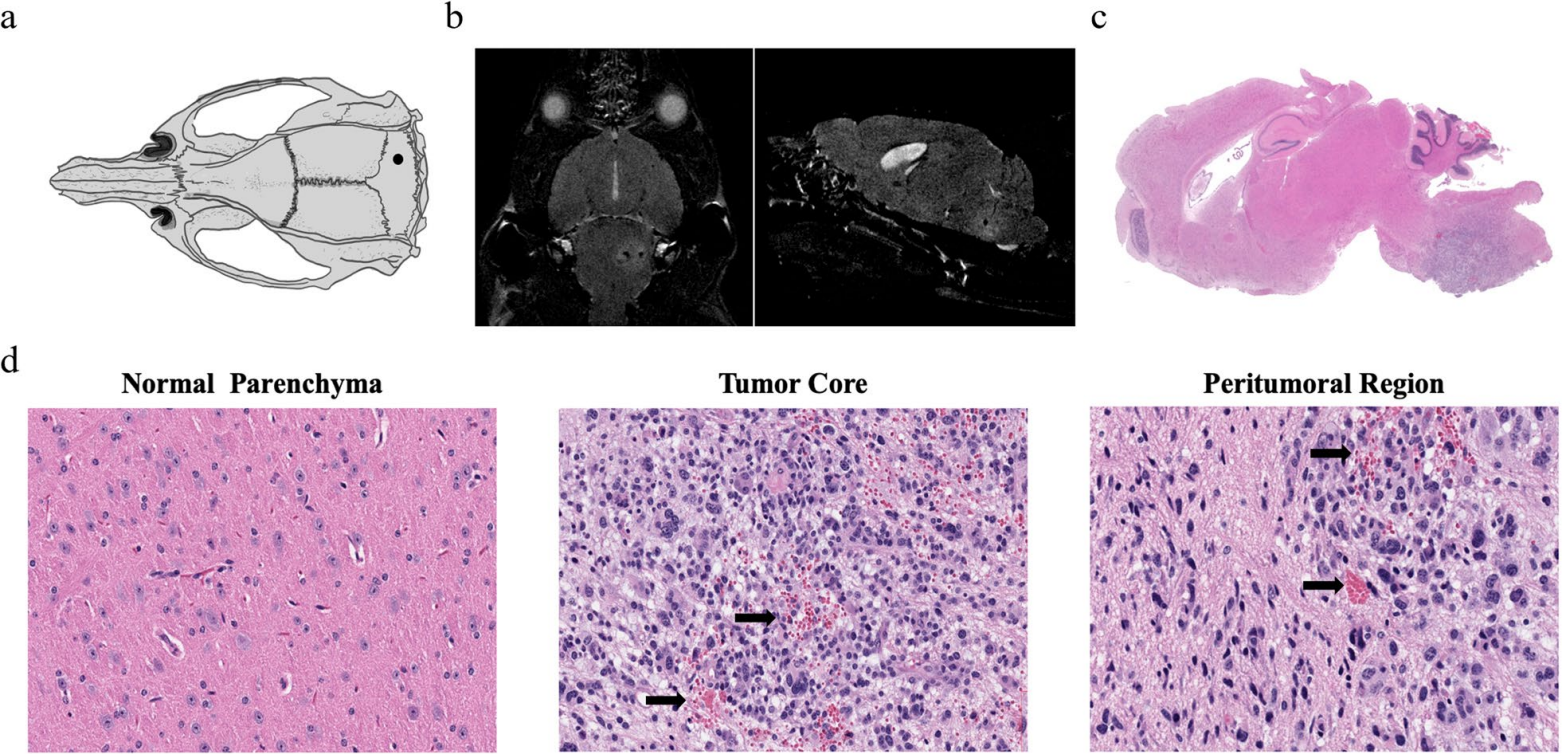
Characterizing a murine syngeneic brainstem DMG model. **a** Intracranial implantation of an H3K27M mutant DMG cell line was achieved by creating a burr hole in the skull at a location posterior to the lambda and lateral to the sagittal suture, with cells injected at a depth of 5.5 mm. **b** Representative images of non-contrast T2-weighted MRI of tumor-bearing mice. **c** Representative photomicrographs of H&E-stained tissue from tumor-bearing mice. **d** Representative H&E staining of normal parenchyma, tumor core, and peritumoral region showing the histopathologic features of murine DMG xenograft. Arrows indicate microhemorrhage

A syngeneic brainstem murine model of DMG was established by intracranial implantation of 4423 DMG cells. The cells were slowly injected via a burr hole in the skull at a location 1.5-mm posterior to the lambda and 1.5-mm lateral to the sagittal suture (Fig. 4a), at a depth of 5.5 mm. We used non-contrast T2 MRI to monitor the tumor growth. The DMG tumor showed hyperintensity on T2-weighted images (Fig. 4b). We analyzed the histopathologic features of the 4423-derived tumor (Fig. 4c). Compared to normal parenchyma, hypercellularity and mitotic features were seen at the tumor core. In the peritumoral region, we observed microscopic disease proliferating from the focal tumor. Tumor core and margins indicated regions of microhemorrhage likely secondary to tumor invasion (Fig. 4d).

**Fig. 5:**
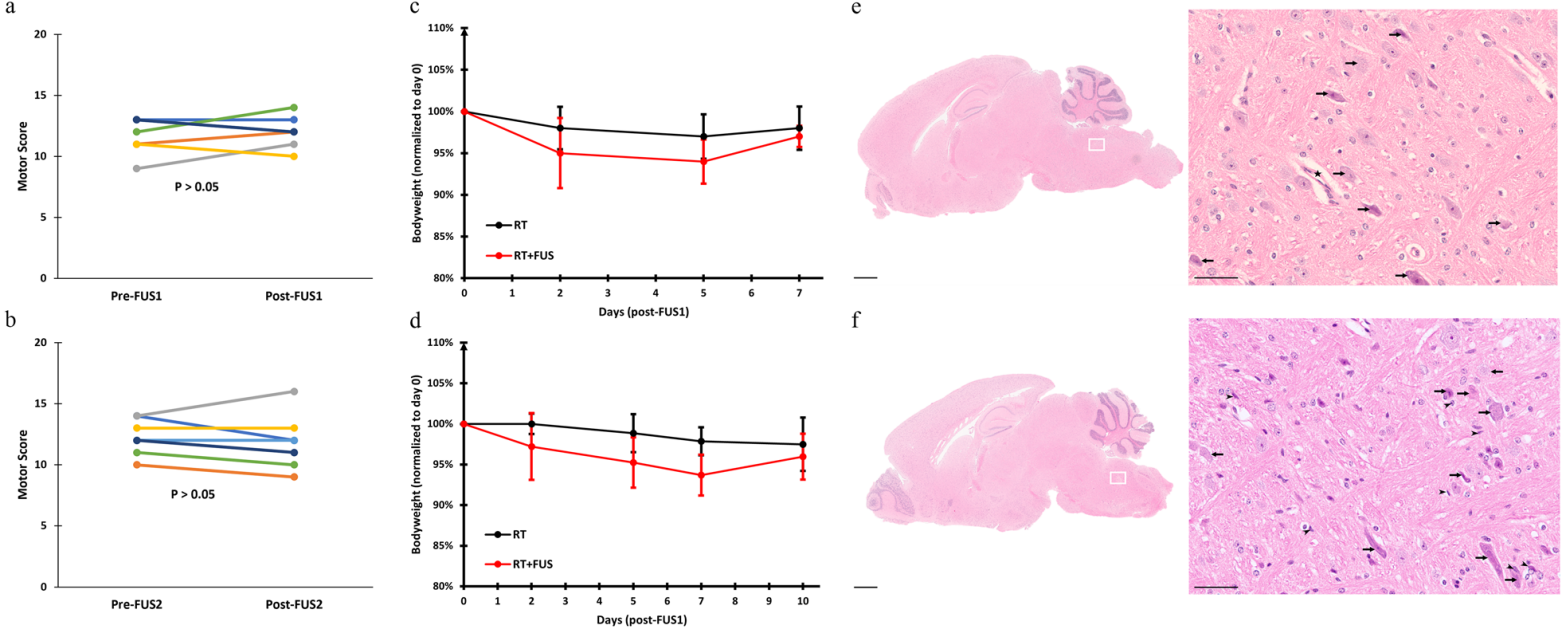
Mice implanted with brainstem DMG tolerated repeated FUS delivery concurrent with RT. Deacon sequential weightlifting test of (**a**) the first and (**b**) the second FUS. Paired motor data for each mouse is represented in different colors for ease of visualization. P-values were calculated by unpaired t tests with Welch’s correction. Body weight curves of RT (black) and RT + FUS (red) animals after (**c**) the first and (**d**) the second FUS. Values are means ± SEM. NS indicates non-significant (P > 0.05) in two-way ANOVA. Representative H&E staining of brainstem DMG mice from (**e**) RT and (**f**) RT + FUS groups. Each right panel is magnified frm the white square of the left panel. Long arrows indicate cell swelling, vacuolar degeneration, and eosinophilic neurons with pyknosis. Arrow heads indicate mononuclear cell infiltration. Filled star: Blood vessel. Scale bar = 1 mm (left panels), 50 µm (right panels)

In all tumor-bearing mice receiving repeated FUS with concurrent RT, no significant difference in motor score was observed before and after each sonication (Fig. 5a, b). Directly following FUS, no significant decline in body weight was observed for RT and RT + FUS mice (Fig. 5c, d). Histological comparison of RT and RT + FUS animals revealed no additional tissue damage and hemorrhage due to repeated brainstem sonication concurrent with RT (Fig. 5e, f). Similar to non-tumor-bearing mice, both groups had mild radiation-induced neuroinflammation and neurodegeneration, including eosinophilic neurons with bright eosinophilic cytoplasm, cellular swelling, and cytoplasmic vacuolation. (Fig. 5e, f, long arrows). The RT + FUS group also had a slight increase in mononuclear cell infiltration (Fig. 5e, f, arrow heads). To identify the specific cell populations infiltrated in brainstem DMG model, we then isolated the mononuclear cells from the brainstem of the tumor-bearing mice and performed flow cytometry analysis (Additional file 1: Fig. S1a). We found that both RT and RT + FUS showed increased trends of microglia compared to untreated tumor-bearing mice, but only the combination group reached statistical significance (Additional file 1: Fig. S1b). Furthermore, the RT + FUS group also showed significant increases in CNS-associated macrophages (Additional file 1: Fig. S1c).

### Effect of RT + FUS combination on survival

**Fig. 6:**
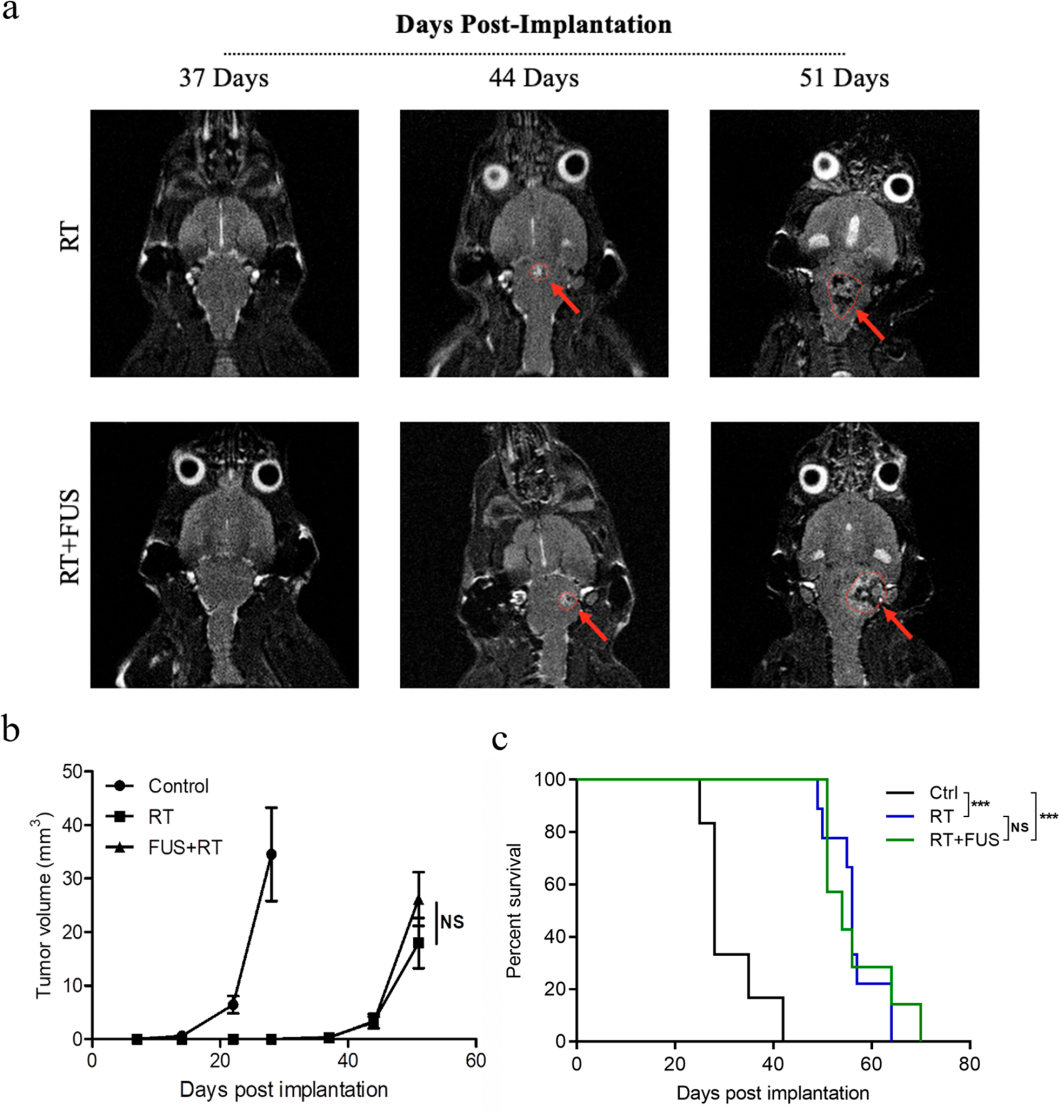
Disease progression kinetics and survival. **a** Representative T2-weighted MRI of RT and RT + FUS mice taken 37, 44, and 51 days following intracranial implantation, with red arrows and outlines indicating tumor presence. **b** Quantitative analysis of the tumor volume of T2-weighted MRI. Values are means ± SEM. NS indicates non-significant (P > 0.05) in two-way ANOVA. **c** Survival analysis. The Kaplan–Meier curve shows the survival of control (black, n = 6), RT (blue, n = 9), and RT + FUS (green, n = 7) animals. *** indicates P < 0.001 and NS indicates non-significant (P > 0.05) using log-rank (Mantel-Cox) test

In our tumor model treated with RT, disease progression occurred in all animals regardless of FUS treatment. T2 MRI-based quantification of tumor volumes for RT and RT + FUS mice after treatment established progression kinetics. In RT and RT + FUS groups, tumor enhancement was seen 44 days following intracranial implantation (21 days for control mice) (Fig. 6a). Compared to the control group, both RT and RT + FUS groups showed much slower tumor growth rates, but no significant difference between RT and RT + FUS groups (Fig. 6b). Kaplan–Meier survival curves showed a statistical increase in survival in both RT (median survival = 56.0 days) and RT + FUS (median survival = 54.0 days) animals, compared to control animals (median survival = 28.0 days) Moreover, median survival times between RT and RT + FUS groups showed no significant difference (Fig. 6c). Collectively, RT exerted both local control and survival benefit in our murine brainstem DMG model. Furthermore, we did not observe increased tumor progression or mortality with combining FUS and RT, indicating repeated FUS-mediated BBBO is well tolerated concurrent with RT.

## Discussion

Brainstem DMG is a fatal pediatric cancer with limited treatment options. Despite decades of research and numerous clinical trials, no effective systemic therapies exist [2]. Surgical resection is not a viable option and RT is the standard-of-care. The BBB remains the major obstacle to potential therapeutic success in this disease, precluding delivery of drugs in sufficient concentrations to the CNS. The brainstem itself offers additional challenges to drug delivery in comparison to other brain areas [30, 31]. Therefore, novel strategies are needed to overcome the BBB.

FUS is an exciting technology that can temporarily disrupt the BBB to enhance drug delivery for a therapeutic benefit without causing local tissue damage. Preclinical studies have shown that FUS can enhance drug delivery by four–eightfold across different CNS disease states [16, 32, 33]. Cerebral primary or secondary tumor studies using FUS have demonstrated local control and increased survival, and several clinical trials evaluating FUS-mediated drug delivery in supratentorial tumors are ongoing [14–22, 34]. However, these efforts are largely limited to adult malignancies, and there is a paucity of investigation regarding safety and feasibility of FUS-mediated BBBO in children, let alone those with brainstem DMG. Select studies demonstrate that FUS delivery to an intact brainstem without tumors can increase drug delivery while preserving tissue integrity and without altering cardiopulmonary or motor function [24, 25]. Using a syngeneic brainstem murine model of DMG, we validated these findings [16]. Such results led to the world’s first pediatric FUS study for children with relapsed DMG [NCT04804709].

Nonetheless, one major limitation for preclinical data to be effectively translated to treating children with upfront DMG is whether brainstem FUS can be safely delivered with RT. Past work demonstrated that adjuvant FUS-mediated BBBO in the supratentorial brain can be achieved safely in animals receiving 30 Gy in 5 fractions as early as 2 days post-treatment [35]. Nonetheless, the safety and feasibility of combining RT and FUS in the brainstem, while receiving clinical doses of RT, had been unknown prior to this study. Radiation regimens for patients with brainstem DMG include 54 Gy in 30 fractions or 39 Gy in 13 fractions [36]. Both regimens show comparable results with improved symptom control and three-month survival benefit, although the latter offers less treatment burden [36]. In this study, we sought to use a clinically relevant RT dose and proceeded with 39 Gy in 13 fractions to facilitate clinical translation of this work.

In phase one of this study, we combined RT and FUS in non-tumor bearing mice to evaluate the safety timeline of combination therapy, advancing the timing of FUS delivery from 1-month after RT, to 1-week after RT, and finally, to concurrently with RT. In phase two, we chose the most aggressive treatment course and utilized a clinically relevant murine syngeneic brainstem DMG model (harboring the H3K27M mutation) to validate these findings [37]. A syngeneic model was chosen to ensure an intact immune system, which is important not only because both RT and FUS have been shown to induce a local sterile inflammatory response, but also to avoid masking potential immune-related toxicities if an immune altered model were used [38, 39]. This also offers the opportunity to use RT + FUS in combination with immunotherapy in future studies.

Throughout this study, we evaluated radiographic, physiological, and histological consequences of concurrent and adjuvant BBBO in mice undergoing hypofractionated RT. In all groups receiving FUS, no permanent differences in heart rate, respiratory rate, motor function, and body weight were noted. No morbidity or mortality was observed when combining RT + FUS. BBBO was confirmed with gadolinium-enhanced T1-weighted MRI, and follow-up MRI at 72-h post-FUS showed BBB closure.

Previous studies have shown that FUS alone may enhance radiosensitivity [40]. However, the addition of FUS during RT did not impact survival. All mice treated with RT had disease progression ~ 44 days after implantation, with no difference in the time, kinetics, or size of progression between RT and RT + FUS mice. This potentially represents an ideal murine model of brainstem DMG to study the effects of FUS-enhanced drug delivery in the setting of RT.

Despite tolerating combined use of RT and FUS, histopathological analysis showed minor neuronal inflammation secondary to RT in all groups. RT has been shown to potentially cause neuroinflammation and neurodegeneration in the brain [41]. However, hypofractionated RT has been proved to be well tolerated and exert similar tumor inhibition in in children with diffuse intrinsic pontine glioma compared to conventionally fractionated RT [6, 36, 42]. Hayashi et al. further reported that the degenerative changes associated with RT were limited from the histology sample of a DMG patient who received hypofractionated RT [42]. From our histological analysis, the addition of FUS did not exacerbate inflammation but increased mononuclear cell infiltration, which is unsurprising given that both RT and FUS are known to have immunomodulatory effects in the CNS [43, 44]. Although we did not perform a further assay to confirm the specific cell type of infiltration in non-tumor-bearing animals, our group recently reported that FUS-mediated BBBO increases microglia and CNS-associated macrophage in the brain [29]. Moreover, our flow cytometry results showed that FUS + RT increased microglia and CNS-associated macrophages in tumor-bearing animals. The potential for combining RT and FUS in the setting of immunotherapy is promising for developing novel treatments. While RT and FUS may modulate the tumor immune microenvironment, FUS may further enhance drug delivery to improve efficacy. Preclinical studies using FUS and immune checkpoint inhibitors have been explored in glioblastoma models [45–47], very little is known regarding trimodality therapy of FUS, RT and immunotherapy. Nonetheless, further studies are needed in the setting of brainstem DMG.

The utility of FUS-mediated drug delivery has garnered much interest since we opened our first clinical trial utilizing FUS and panobinostat for children with relapsed DMG [NCT04804709] [48, 49]. Since then, there have been two additional phase I clinical trials opened using FUS and doxorubicin for children with newly diagnosed DMG [NCT05630209; NCT05615623]. In addition, we opened our second Phase I study utilizing etoposide with FUS for patients with relapsed DMG [NCT05762419]. As the clinical interest for FUS continues to grow, there will be a need to combine potential radiation sensitizing drugs with FUS-mediated drug delivery. Our findings are critical for designing the next phase of clinical trials using FUS and RT in patients with DMG. Nonetheless, there are a few limitations to be considered. These include a need to better characterize the tumor immune microenvironment and understand how combinatorial RT and FUS can be leveraged for immunotherapeutic applications. In addition, the new classification of DMG suggests that thalamic and other midline brain tumors harboring the H3K27M mutation may benefit from FUS. Safety and feasibility in these structures are also currently under investigation.

## Conclusion

This is the first study to demonstrate that repeated FUS-mediated BBBO is safe and feasible with a concurrent hypofractionated clinical dose of RT to the brainstem for DMG. This is a critical finding for next steps of Phase I clinical trial planning of FUS studies in patients with DMG combining this treatment paradigm with standard-of-care radiotherapy.

### Supplementary Information

**Additional file 1: Figure S1. **Flow cytometric analysis of microglia and CNS-associated macrophage in murine DMG tumors. **(a)** Representative images of gating strategy used in flow cytometric analyses. The microglia were identified by CD45^low^ CD11b^+^ CX3CR1^+^ population. The CNS-associated macrophages were identified by CD45^high^ CD11b^+^ CD80^+^ CD86^+^ population. Quantitation of **(b)** microglia and **c)** CNS-associated macrophages infiltrated in murine DMG tumors upon different treatments. Values are means + SEM; ∗ indicates a P value < 0.05 in an unpaired t test with Welch's correction, compared with the control group.

## Data Availability

Research data are stored in an institutional repository and will be shared upon request to the corresponding author.

## Abbreviations

DMG: Diffuse midline glioma
RT: Radiotherapy
BBB: Blood–brain barrier
CNS: Central nervous system
39 Gy/13fx: 39 Gy in 13 fractions
FUS: Focused ultrasound
BBBO: Blood–brain barrier opening
SARRP: Small animal radiation research platform
MRI: Magnetic resonance imaging
MBs: Microbubbles
PCD: Passive cavitation detection
SCDh: Stable harmonic cavitation dose
SCDu: Stable ultraharmonic cavitation dose
ICD: Inertial cavitation dose
T1 + C: T1-weighted contrast-enhanced
H&E: Hematoxylin and eosin
