# Supplementary material for: Focused ultrasound-mediated blood–brain barrier opening is safe and feasible with moderately hypofractionated radiotherapy for brainstem diffuse midline glioma
Source: J Transl Med. 2024 Mar 30;22:320. doi: 10.1186/s12967-024-05096-9 (PMC10981822; doi:10.1186/s12967-024-05096-9)
Supplement: Supplementary file 1 — Additional file 1: Figure S1. Flow cytometric analysis of microglia and CNS-associated macrophage in murine DMG tumors. (a) Representative images of gating strategy used in flow cytometric analyses. The microglia were identified by CD45low CD11b+ CX3CR1+ population. The CNS-associated macrophages were identified by CD45high CD11b+ CD80+ CD86+ population. Quantitation of (b) microglia and c) CNS-associated macrophages infiltrated in murine DMG tumors upon different treatments. Values are means + SEM; ∗ indicates a P value < 0.05 in an unpaired t test with Welch's correction, compared with the control group. [file 12967_2024_5096_MOESM1_ESM.docx]

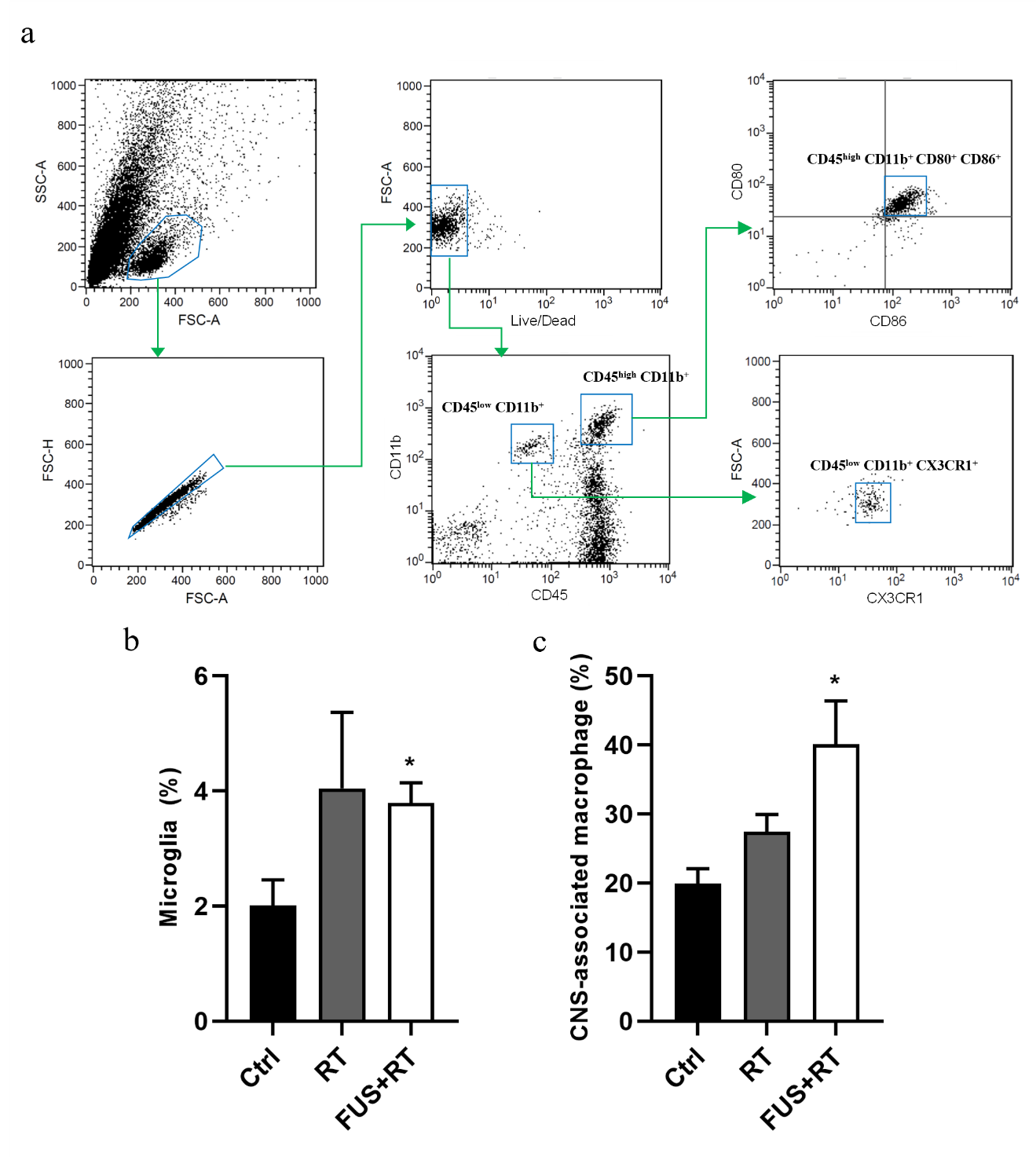


**Figure S1.** Flow cytometric analysis of microglia and CNS-associated macrophage in murine DMG tumors. **a)** Representative images of gating strategy used in flow cytometric analyses. The microglia were identified by CD45^low^ CD11b^+^ CX3CR1^+^ population. The CNS-associated macrophages were identified by CD45^high^ CD11b^+^ CD80^+^ CD86^+^ population. Quantitation of **b)** microglia and **c)** CNS-associated macrophages infiltrated in murine DMG tumors upon different treatments. Values are means + SEM; ∗ indicates a P value < 0.05 in an unpaired t test with Welch's correction, compared with the control group.
